# New Entropic Inequalities and Hidden Correlations in Quantum Suprematism Picture of Qudit States †

**DOI:** 10.3390/e20090692

**Published:** 2018-09-11

**Authors:** Margarita A. Man’ko, Vladimir I. Man’ko

**Affiliations:** 1 Lebedev Physical Institute, Russian Academy of Sciences, Leninskii Prospect 53, Moscow 119991, Russia; 2 Moscow Institute of Physics and Technology (State University), Institutskii per. 9, Dolgoprudnyi, Moscow Region 141700, Russia; 3 Department of Physics, Tomsk State University, Lenin Avenue 36, Tomsk 634050, Russia

**Keywords:** entropy, correlations, qubits, probability representation, Bayes’ formula

## Abstract

We study an analog of Bayes’ formula and the nonnegativity property of mutual information for systems with one random variable. For single-qudit states, we present new entropic inequalities in the form of the subadditivity and condition corresponding to hidden correlations in quantum systems. We present qubit states in the quantum suprematism picture, where these states are identified with three probability distributions, describing the states of three classical coins, and illustrate the states by Triada of Malevich’s squares with areas satisfying the quantum constraints. We consider arbitrary quantum states belonging to *N*-dimensional Hilbert space as $\left(N^{2}-1\right)$ fair probability distributions describing the states of $\left(N^{2}-1\right)$ classical coins. We illustrate the geometrical properties of the qudit states by a set of Triadas of Malevich’s squares. We obtain new entropic inequalities for matrix elements of an arbitrary density *N*×*N*-matrix of qudit systems using the constructed maps of the density matrix on a set of the probability distributions. In addition, to construct the bijective map of the qudit state onto the set of probabilities describing the positions of classical coins, we show that there exists a bijective map of any quantum observable onto the set of dihotomic classical random variables with statistics determined by the above classical probabilities. Finally, we discuss the physical meaning and possibility to check derived inequalities in the experiments with superconducting circuits based on Josephson junction devices.

## 1. Introduction

Generic states of quantum systems are identified with the density matrices [1,2] or the density operators $\hat{\rho }$ acting in a Hilbert space. The pure states of quantum systems are identified with the state vectors ∣ψ〉 belonging to the Hilbert space [3] and complex wave functions [4,5] ψ(x)=〈x∣ψ〉, where *x* is an observable, e.g., the continuous position of a particle. The physical meaning of the wave function ψ(x) is related to measuring the observable *x*; in the state ∣ψ〉, the measurement of the position of a particle yields the probability density ${\left|\langle x|\psi \rangle \right|}^{2}={\left|\psi \left(x\right)\right|}^{2}$, which does not contain information on the phase of the complex wave function.

For spin-*s* systems with discrete observables like spin projections m=−s,−s+1,…,s−1,s; s=0,1/2,1,3/2,2,…, the state vectors belong to the Hilbert space of finite dimension N=2s+1, and the complex wave function ψ(m)=〈m∣ψ〉 determines the probability distribution ${\left|\langle m|\psi \rangle \right|}^{2}={\left|\psi \left(m\right)\right|}^{2}$ associated with the state ∣ψ〉. The phase of the wave function is not determined by the probability distribution; in view of this fact, information on the state ∣ψ〉, contained in the probability density ${\left|\psi \left(x\right)\right|}^{2}$ or in the probability distribution ${\left|\psi \left(m\right)\right|}^{2}$, is not sufficient to describe the particle’s pure state or the spin-*s* pure state.

The aim of this paper is to consider the old problem of looking for a such formulation of quantum mechanics, where the system states can be identified with fair probability distributions of measurable observables only. Such a possibility is based on quantum tomography methods of measuring [6] quantum states, using the formalism of reconstructing [7,8] the state Wigner function by means of Radon transform [9].

Wigner introduced the Wigner function [10] W(q,p) of the position *q* and momentum *p* that is similar to the probability density f(q,p) describing the classical particle state in the presence of fluctuations. The Wigner function can take negative values and, due to this circumstance, it is called the quasidistribution function. The Wigner function is related to the density matrix $\rho (x,x^{\prime })$ of the quantum particle state by an invertible Fourier transform and contains the same information on the state as the density matrix.

There exist other analogous quasidistributions like the Husimi–Kano *Q*-function [11,12] and Glauber–Sudarshan function [13,14], which are functions on the phase space. The suggestion to identify quantum states with fair probability densities was presented in [15], where the probability density, called symplectic tomogram, was used. An analogous approach was elaborated for spin states in [16,17], where the spin tomograms, being fair probability distributions of spin projections *m* on an arbitrary direction in the space given by a unit vector $\vec{n}$, were shown to determine the density matrix $\rho (m,m^{\prime })$.

In this paper, on the example of qubits, we show the bijective map of density operators of spin-1/2 states onto the probability distributions. Since for probability distributions the notion of Shannon entropy [18], relative entropy, and Tsallis entropy [19] is the standard tool to characterize the statistical properties of the systems, we obtain, in view of the map introduced, some new relations like entropic equalities and inequalities for quantum spin states. Other kinds of entropies also exist like Rényi entropy [20], non-Shannonian and generalized (c,d) entropies; see, e.g., [21,22]. In this paper, we consider new relations connected with Shannon and Tsallis entropies. In addition, we discuss new geometric interpretation of spin-1/2 (qubit) states in terms of the Triada of Malevich’s squares [23,24] and its relation to the Bloch sphere geometry of these states. Employing the identification of qubit states with probability distributions, we present the construction of quantum observables (Hermitian 2 × 2-matrices) in terms of sets of classical-like variables and provide the bijective map of the qubit states (density matrices) and observables onto classical-coin probability distributions and classical observables associated with these coins.

We present the evolution equations for the density matrices of qubit states in the form of kinetic equations for probability distributions determining the qubit states. We formulate the superposition principle of qubit state vectors as a new addition rule for the probabilities determining the states. In addition, we express the Born rule for calculating the probability ${\left|\langle \psi_{1}|\psi_{2}\rangle \right|}^{2}=w_{12}$ as a function of probabilities determining the pure states $|\psi_{1}\rangle$ and $|\psi_{2}\rangle$. Then, we extend the probability representation of qubit states and express the matrix elements of an arbitrary density *N*×*N*-matrix in terms of classical-coin probability distributions. We consider in detail examples of qutrit (spin-1), identifying the qutrit state with a set of Triadas of Malevich’s squares. We present new relations of areas of Malevich’s squares and the possibility of checking these relations in the experiments with superconducting circuits.

The other goal of this work is to study within the probability representation of quantum states [15,16,17,25,26,27,28] (reviewed in [29]) the triangle geometry of qudit states and discuss Bayes’ formula for systems without subsystems and correlations (called the hidden correlations) in such systems. It is worth noting that the classical probability distributions were discussed within the framework of state vectors for spin-1/2 systems by Khrennikov [30,31,32] and the superposition principle for spin-1/2 states was expressed as the nonlinear superposition of classical probability distributions in [33,34]. Malevich’s squares and the approach called the suprematism in art are described in [35].

This paper is organized as follows.

We present the notion of random variables in Section 2 and study Bayes’ formula for systems with one random variable in Section 3. We discuss qubit states in Section 4 and consider classical-coin random variables for qubit systems in Section 5. Then, we review the notion of quantum suprematism in Section 6 and study qutrit states in the probability representation in Section 7. We devote Section 8 to the superposition principle for the probabilities, demonstrating this principle on the example of qutrits. Within the framework of the probability representation, we formulate the superposition principle for qudit states in Section 9. Finally, in Section 10, we provide the conclusions and perspectives.

## 2. Random Variables and Probabilities

In probability theory, the notion of random variables and probability distributions were discussed using rigorous approaches presented, for example, in [36,37,38,39]. We employ here the following empiric approach. We define the relation of random variables to sets of integer numbers following [40,41]. Given a set of *N* different events, these events are associated with integers j=1,2,…,N. We call relative frequencies P(j) of the realization of these random events in a series of experiments “the probabilities of the events” where 0≤P(j)≤1. The function P(j) is the probability distribution; it is normalized $\sum_{j=1}^{N}P\left(j\right)=1$.

The properties of the events are characterized by some functions f(j), which we call observables. In this approach, random variables are mapped onto the integers j=1,2,…,N. The physical meaning of the events can be different; for example, in the casino roulette, the event is the appearance of some integer number *j* which is chosen from a set of integer numbers located between 1 and *N*. The event may be also considered as positions “UP” and “DOWN” of two coins; in this case, the integer number *j* is mapped onto a pair (a,b) of integer numbers labeling the position of each coin. In both cases, the relative frequencies of the events can be associated with the integer *j*, but the interpretation of this random variable is different. In the case of casino roulette, we say about one random variable, and in the case of two coins, we have two random variables associated with labeling positions of two coins by other two integer numbers (a,b). An analogous approach to random events can be employed in quantum mechanics.

We extend the above approach to classical probabilities using in this case the identification of random events with the integers $1\leq j,j^{\prime }\leq N$ labeling the matrix elements of the density matrix $\rho_{jj^{\prime }}$ determining the states, e.g., of qudit with spin *s*, where N=2s+1, or of the *N*-level atom. The physical observables are given by the Hermitian matrices $f_{jj^{\prime }}$, where indices of rows and columns are identified with the random variables $1\leq j,j^{\prime }\leq N$. It is important that we can interpret the described above association of integers *j* analogously to the case of classical casino roulette and the case of two classical coins considering numerically the same density matrices $\rho_{jj^{\prime }}$ either as the density matrices of noncomposite (nondivisible) systems (an analog of the casino roulette) or as the density matrices of bipartite systems (an analog of the states of two coins).

In the next section, we consider Bayes’ formula, in view of the approach under discussion, using it for one random variable and applying the map of integer numbers 1,2,…,N onto pairs of random numbers.

## 3. Bayes’ Formula for the Probability Distribution of One Random Variable

In this section, we discuss the application of Bayes’ formula available for probability distributions of several random variables to the case of the probability distribution of one random variable.

First, we recall Bayes’ formula and the notion of conditional probability distribution for statistics of two random variables. Given the function 1≥P(j,k)≥0, where $j=1,2,\ldots ,n_{1}$, $k=1,2,\ldots ,n_{2}$, and $n_{1}n_{2}=N$, with the normalization condition

(1) $$\sum_{j=1}^{n_{1}}\sum_{k=1}^{n_{2}}P\left(j,k\right)=1.$$

This function is identified with the probability distribution of two random variables *j* and *k*. The marginal probability distributions

(2) $$P_{1}\left(j\right)=\sum_{k=1}^{n_{2}}P\left(j,k\right),\;P_{2}\left(k\right)=\sum_{j=1}^{n_{1}}P\left(j,k\right)$$

determine the statistical properties of each random variable.

The conditional probability distribution of the first random variable *j* for given *k* is presented by the formula; see [36],

(3) $$P\left(j|k\right)=\frac{P(j,k)}{P_{2}\left(k\right)}\;,$$

which means that

(4) $$P\left(j,k\right)=P_{2}\left(k\right)P\left(j|k\right).$$

For the case of joint probability distributions of two random variables describing the statistics of the bipartite system, these relations correspond to Bayes’ formula connecting marginal probability distributions and conditional probability distributions of these random variables. In Appendix A, we present an example of application of the above formulas for a particular case N=4. In view of the example from Appendix A, we are in the position to formulate the rule for introducing Bayes’ formula for the probability distribution P(n); $N=n_{1},n_{2}$ of one random variable. We apply the map of integers *n* onto pairs of integers *j* and *k*, such that $j=1,2,\ldots ,n_{1}$ and $k=1,2,\ldots ,n_{2}$. Then, for marginal probability distributions and conditional probability distributions, we use the known expression for joint probability distribution of two variables and define these distributions, in view of the invertible map of integers $1,2,\ldots ,N↔\left(1,1\right),\left(2,1\right),\ldots ,\left(n_{1},1\right)\left(1,2\right),\left(2,2\right),\ldots ,\left(n_{1},2\right),\ldots ,\left(n_{1},n_{2}\right)$, where $N=n_{1}n_{2}$. This map can be described by the functions discussed in [42,43,44].

Following [44], we determine the functions $y(x_{1},x_{2})$, $x_{1}\left(y\right)$, and $x_{2}\left(y\right)$, where $1\leq x_{1}\leq X_{1}$, $1\leq x_{2}\leq X_{2}$, and $1\leq y\leq N=X_{1}X_{2}$, as

(5) $$\begin{array}{c} y\left(x_{1},x_{2}\right)=x_{1}+\left(x_{2}-1\right)X_{1}, \end{array}$$

(6) $$\begin{array}{c} x_{1}\left(y\right)=y\;\mathrm{mod}\;X_{1},\;1\leq y\leq N, \end{array}$$

(7) $$\begin{array}{c} x_{2}\left(y\right)-1=\frac{y-x_{1}\left(y\right)}{X_{1}}\;\mathrm{mod}\;X_{2},\;1\leq y\leq N. \end{array}$$

We use these functions for representing the probability distribution of one random variable as a joint probability distribution of two random variables. To do this, we introduce in Equations (5)–(7) the following notation: y≡n, $x_{1}\equiv j$, $x_{2}\equiv k$, $X_{1}\equiv n_{1}$, $X_{2}\equiv n_{2}$, $N=n_{1}n_{2}=X_{1}X_{2}$, n=1,2,…,N, $P\left(j,k\right)\equiv y\left(x_{1},x_{2}\right)$, and f(y)=P(n). In the case of N=4 and $n_{1}=n_{2}=2$, the map introduced just provides the relations P(1)=P(1,1), P(2)=P(2,1), P(3)=P(1,2), and P(4)=P(2,2) discussed above. Nevertheless, the functions introduced describe the invertible map of the probability distribution of one random variable P(n); n=1,2,…,N onto the joint probability distribution P(j,k) of two random variables $j=1,2,\ldots ,n_{1}$ and $k=1,2,\ldots ,n_{2}$, with $N=n_{1}n_{2}$, for arbitrary integers $n_{1}$ and $n_{2}$. In our new notation, n=n(j,k), j=j(n), and k=k(n). Taking into account this discussion, we introduce Bayes’ formula for the probability distribution P(n) of one random variable; it reads

(8) $$P\left(j(n)|k(n)\right)=\frac{P\left(n(j,k)\right)}{\sum_{j=1}^{n}P\left(n(j,k)\right)},\;n=n_{1}n_{2},$$

where functions n(j,k), j(n), and k(n) are constructed in [44].

The relation of the joint probability distribution P(j,k) to the marginal probability distributions corresponds to the presence of correlations in the system with two random variables. Since we introduced an analog of two random variables and their marginal and conditional probability distributions, the relation of these distributions reflect correlations, which we called [45] the hidden correlations for systems without subsystems. Such correlations exist for both classical and quantum systems.

Bayes’ formula can also be considered for the probability distribution of one random variable P(n), if the integer n=1,2,…,N and $N=n_{1}n_{2}n_{3}$, where $n_{1}$, $n_{2}$, and $n_{3}$ are integers of the joint probability distribution of three random variables P(j,k,l), with $j=1,2,\ldots ,n_{1}$, $k=1,2,\ldots ,n_{2}$, and $l=1,2,\ldots ,n_{3}$. To do this, we use an analogous invertible map [44] of functions $y(x_{1},x_{2},x_{3})$, $x_{1}\left(y\right)$, $x_{2}\left(y\right)$, and $x_{3}\left(y\right)$, taking integer values $y=1,2,\ldots ,N=X_{1}X_{2}X_{3}$; $1\leq x_{i}\leq X_{i}$; i=1,2,3, defined by the relations

(9) $$\begin{array}{c} y\left(x_{1},x_{2},x_{3}\right)=x_{1}+\left(x_{2}-1\right)X_{1}+\left(x_{3}-1\right)X_{1}X_{2}, \end{array}$$

(10) $$\begin{array}{c} x_{1}\left(y\right)=y\;\mathrm{mod}\;X_{1}, \end{array}$$

(11) $$\begin{array}{c} x_{2}\left(y\right)-1=\frac{y-x_{1}\left(y\right)}{X_{1}}\;\mathrm{mod}\;X_{2}, \end{array}$$

(12) $$\begin{array}{c} x_{3}\left(y\right)-1=\frac{y-x_{1}\left(y\right)-x_{2}\left(y\right)X_{1}}{X_{1}X_{2}}\;\mathrm{mod}\;X_{3}. \end{array}$$

After substitution $x_{1}\equiv j$, $x_{2}\equiv k$, $x_{3}\equiv l$, and y≡n, we arrive at an analog of Bayes’ formula for one random variable

(13) $$P\left(j(n)|k(n),l(n)\right)=\frac{P\left(j(n),k(n),l(n)\right)}{\sum_{j=1}^{n_{1}}P\left(n(j,k,l)\right)}.$$

To illustrate this formula, we consider the example of N=8=2·2·2, i.e., $n_{1}=n_{2}=n_{3}=2$; the map of integers reads 1↔(1,1,1), 2↔(2,1,1), 3↔(1,2,1), 4↔(2,2,1), 5↔(1,1,2), 6↔(2,1,2), 7↔(1,2,2), 8↔(2,2,2). This means that the probability distribution P(n) takes the values P(1)≡P(1,1,1), P(2)≡P(2,1,1), P(3)≡P(1,2,1), P(4)≡P(2,2,1), P(5)≡P(1,1,2), P(6)≡P(2,1,2), P(7)≡P(1,2,2), and P(8)≡P(2,2,2). The joint probability distribution P(j,k,l) has the values given by numbers P(n), and Bayes’ formula obtained provides, e.g., the conditional probability

$$P\left(j(n)=1|k(n)=1,l(n)=1\right)=\frac{P(1,1,1)}{P(1,1,1)+P(2,1,1)}=\frac{P(1)}{P(1)+P(2)}\;.$$

In the quantum case, the map of integers discussed provides a tool to consider the density matrix of qudit state $\rho_{nn^{\prime }}$, where $n,n^{\prime }=1,2,\ldots ,N$, as the density matrix of a multipartite system. For example, at N=4, the ququart density matrix can be interpreted as the density matrix of two two-level atoms, using the map discussed. In fact, if $n,n^{\prime }=1,2,3,4$, we consider the density matrix as $\rho_{nn^{\prime }}\equiv \rho_{jk,j^{\prime }k^{\prime }}$, where $j,j^{\prime },k,k^{\prime }=1,2$. Formally, we obtain the density matrix of the two-qubit system, which has the same numerical matrix elements that the 4×4-matrix $\rho_{nn^{\prime }}$; this means that all numerical properties of the density matrix of two-qubit state and ququart state are identical.

This fact provides the possibility to consider formal entanglement properties of ququart system. For example, if we consider the pure state, ${\left(\rho^{2}\right)}_{nn^{\prime }}=\rho_{nn^{\prime }}$, then the properties of linear entropy $S=1-Tr\;\left(\rho^{2}\left(1\right)\right)$, with ${\left(\rho (1)\right)}_{jj^{\prime }}=\sum_{k=1}^{2}\rho_{jk,\;j^{\prime }k}$, where the indices $j,k,j^{\prime }$ are determined by the numbers $n,n^{\prime }=1,2,3,4$ according to the discussed map, characterize the entanglement degree in the bipartite system.

For the four-level atom, one has the same numerical characteristics. From the viewpoint of the matrix properties, the ququart state with the density matrix, having only different from zero matrix elements $\rho_{11}=\rho_{14}=\rho_{41}=\rho_{44}=1/2$, provides the linear entropy S=1/2 corresponding to maximum entangled state of two qubits. The interpretation of this phenomenon for systems without subsystems is the presence of hidden correlations in the degrees of freedom of such systems, formally analogous to quantum correlations associated with the entanglement phenomenon, e.g., in bipartite systems of two qubits.

## 4. Probability Representation of Spin-1/2 States

We start our introduction of the probability representation of quantum system states with the consideration of spin-1/2 systems. These systems realize qubits and their states, as well as they are realized by two-level atom systems. In standard formulation of quantum mechanics, the spin-1/2 pure states are described by Pauli spinors, which are complex vectors ∣ψ〉 with two components, i.e., $|\psi \rangle =\left(\begin{array}{c} \varphi_{1}\\ \varphi_{2} \end{array}\right).$ The vectors ∣ψ〉 belong to the two-dimensional Hilbert space H with the scalar product

(14) $$\langle \psi^{\left(1\right)}|\psi^{\left(2\right)}\rangle =\varphi_{1}^{(1)*}\varphi_{1}^{\left(2\right)}+\varphi_{2}^{(1)*}\varphi_{2}^{\left(2\right)}.$$

The state vectors are normalized 〈ψ∣ψ〉=1, and ${\left|\varphi_{1}\right|}^{2}+{\left|\varphi_{2}\right|}^{2}=1.$ The density operators [1,2] of the pure states $\rho_{\psi }=|\psi \rangle \langle \psi |$ in matrix form read $\rho_{\psi }=\left(\begin{array}{cc} \varphi_{1}^{*}\varphi_{1} & \varphi_{1}^{*}\varphi_{2}\\ \varphi_{2}^{*}\varphi_{1} & \varphi_{2}^{*}\varphi_{2} \end{array}\right).$ This matrix has the properties of Hermiticity $\rho_{\psi }^{†}=\rho_{\psi }$ and nonnegativity $\rho_{\psi }\geq 0$, as well as it has the unit trace Tr $\rho_{\psi }=1$.

The physical meaning of the state-vector components $\varphi_{1}$ and $\varphi_{2}$ and matrix elements of the density matrix ρ(ψ) is determined by the relation of these values to operators of physical observables associated with spin projection operators $\hbar \sigma_{x}/2$, $\hbar \sigma_{y}/2$, and $\hbar \sigma_{z}/2$ onto the axes *x*, *y*, and *z*, respectively. Here, the Pauli matrices $\sigma_{x}$, $\sigma_{y}$, and $\sigma_{z}$ are

(15) $$\sigma_{x}=\left(\begin{array}{cc} 1 & 1\\ 1 & 0 \end{array}\right),\;\sigma_{y}=\left(\begin{array}{cc} 0 & -i\\ i & 0 \end{array}\right),\;\sigma_{z}=\left(\begin{array}{cc} 1 & 0\\ 0 & -1 \end{array}\right),$$

and ℏ is the Planck constant. In this paper, we use dimensionless units and assume ℏ=1. Three normalized eigenstates of the matrices $\sigma_{x}/2$, $\sigma_{y}/2$, and $\sigma_{z}/2$ with eigenvalues +1/2 have the form

(16) $$|\psi_{x}\rangle =\frac{1}{\sqrt{2}}\left(\begin{array}{c} 1\\ 1 \end{array}\right),\;|\psi_{y}\rangle =\frac{1}{\sqrt{2}}\left(\begin{array}{c} 1\\ i \end{array}\right),\;|\psi_{z}\rangle =\left(\begin{array}{c} 1\\ 0 \end{array}\right).$$

The vectors are identified with spin-1/2 states, in which the spin projections on the axes *x*, *y*, and *z* are equal to +1/2. The corresponding density matrices read

(17) $$|\psi_{x}\rangle \langle \psi_{x}|=\left(\begin{array}{cc} 1/2 & 1/2\\ 1/2 & 1/2 \end{array}\right),\;|\psi_{y}\rangle \langle \psi_{y}|=\left(\begin{array}{cc} 1/2 & -i/2\\ i/2 & 1/2 \end{array}\right),\;|\psi_{z}\rangle \langle \psi_{z}|=\left(\begin{array}{cc} 1 & 0\\ 0 & 0 \end{array}\right).$$

Any density matrix describing mixed state of the spin-1/2 system $\rho =\left(\begin{array}{cc} \rho_{11} & \rho_{12}\\ \rho_{21} & \rho_{22} \end{array}\right)$, such that $\rho^{†}=\rho$, Tr ρ=1, and ρ≥0 (i.e., the matrix has nonnegative eigenvalues), is determined by three real parameters. The physical meaning of these parameters can be clarified, if one considers the probabilities to obtain in the state with the density matrix ρ the spin projections +1/2 on the axes *x*, *y*, *z*, which we denote as $p_{1}$, $p_{2}$, and $p_{3}$, respectively. The probabilities $p_{1}$, $p_{2}$, and $p_{3}$ play a fundamental role in describing the spin-1/2 states and, as we show, they determine the density matrix of this system. These probabilities are given by the Born rule as follows:

(18) $$p_{1}=Tr\left(\rho |\psi_{x}\rangle \langle \psi_{x}|\right),\;p_{2}=Tr\left(\rho |\psi_{y}\rangle \langle \psi_{y}|\right),\;p_{3}=Tr\left(\rho |\psi_{z}\rangle \langle \psi_{z}|\right).$$

The spin tomogram $w(m|\vec{n})$ introduced in [16,17], being equal to the conditional probability of spin projection m=±1/2 onto the direction given by the unit vector $\vec{n}$, is expressed in terms of the probability vector $\vec{p}=\left(p_{1},p_{2},p_{3}\right)$ [23], i.e.,

(19) $$w\left(m|\vec{n}\right)=\left(1/2\right)+m\left(\vec{p}-{\vec{p}}_{0}\right)\vec{n},\;{\vec{p}}_{0}=\left(1/2,1/2,1/2\right).$$

In view of relations (18) employed as the equations for matrix elements of ρ, it is not difficult to rewrite the density matrix ρ in the form where its matrix elements are expressed in terms of the probabilities $p_{1}$, $p_{2}$, and $p_{3}$ [23,24,46,47]; we have

(20) $$\rho =\left(\begin{array}{cc} p_{3} & p_{1}-\left(1/2\right)-i\left(p_{2}-1/2\right)\\ p_{1}-\left(1/2\right)+i\left(p_{2}-1/2\right) & 1-p_{3} \end{array}\right).$$

The standard parameters of the Bloch sphere of qibit states $x_{1}$, $x_{2}$, and $x_{3}$ are connected with the probabilities through the bijective map $x_{k}=2p_{k}-1$; k=1,2,3.

If the spin-1/2 state is the pure state, its density matrix satisfies the constraint $\rho^{2}=\rho$ that provides the condition for probabilities

(21) $${\left(p_{1}-1/2\right)}^{2}+{\left(p_{2}-1/2\right)}^{2}=p_{3}\left(1-p_{3}\right).$$

In this case, the Pauli spinor of the pure state ∣ψ〉 can also be expressed in terms of the three probabilities satisfying condition (21), i.e.,

(22) $$|\psi \rangle =\left(\begin{array}{c} \sqrt{p_{3}}\\ \frac{p_{1}-1/2}{\sqrt{p_{3}}}+i\;\frac{p_{2}-1/2}{\sqrt{p_{3}}} \end{array}\right).$$

For mixed states, the nonnegativity condition of the density matrix (nonnegativity condition for its eigenvalues) yields the inequality for the probabilities $p_{1}$, $p_{2}$, and $p_{3}$; it reads

(23) $${\left(p_{1}-1/2\right)}^{2}+{\left(p_{2}-1/2\right)}^{2}+{\left(p_{3}-1/2\right)}^{2}\leq 1/4.$$

As we see, all information on the spin-1/2 state density matrix (and its Pauli spinor describing the pure state ∣ψ〉) is identified with three probabilities $0\leq p_{1},p_{2},p_{3}\leq 1$ satisfying inequality (23).

This observation provides the possibility to consider again very old problem of quantum mechanics, namely: Is it possible to formulate the notion of quantum states employing only ingredients of classical probability theory of systems with fluctuations, such as the probability distributions?

We observed that for spin-1/2 systems it is enough to have three probability distributions given by the probability vectors ${\vec{P}}_{1}=\left(\begin{array}{c} p_{1}\\ 1-p_{1} \end{array}\right)$, ${\vec{P}}_{2}=\left(\begin{array}{c} p_{2}\\ 1-p_{2} \end{array}\right)$, and ${\vec{P}}_{3}=\left(\begin{array}{c} p_{3}\\ 1-p_{3} \end{array}\right).$ Inequality (23) is the only one quantum condition which should be respected by the probabilities. Thus, instead of vectors ∣ψ〉 and density matrices ρ, we can introduce the notion of spin-1/2 states, employing the set of three probability distributions or identify the state with the vector $\vec{P}=\left(\begin{array}{c} p_{1}\\ p_{2}\\ p_{3} \end{array}\right)$. This means that all quantum phenomena like, e.g., quantum interference, can be described in terms of the probabilities. Here, it worth noting that the interference of classical probabilities was discussed in [30,31,32]. For example, the superposition principle of quantum states, expressed in terms of normalized and orthogonal state vectors $|\psi_{1}\rangle$ and $|\psi_{2}\rangle$ by the equality

(24) $$|\psi \rangle =\sqrt{\Pi_{3}}|\psi_{1}\rangle +\sqrt{1-\Pi_{3}}\;e^{i\xi }|\psi_{2}\rangle ,$$

where ∣ψ〉 is again the state vector, can be formulated as “superposition” of probabilities.

We present the result in the form of a nonlinear addition of two vectors

(25) $${\vec{P}}^{\left(1\right)}⊕{\vec{P}}^{\left(2\right)}={\vec{P}}^{\left(3\right)},$$

where components of the vectors are the probabilities $p_{1}^{\left(k\right)}$, $p_{2}^{\left(k\right)}$, and $p_{3}^{\left(k\right)}$; k=1,2,3, satisfying equality (21) for each value of *k*. The notation of addition ⊕ is also associated with the probability vector $\vec{\Pi }=\left(\begin{array}{c} \Pi_{1}\\ \Pi_{2}\\ \Pi_{3} \end{array}\right)$, where the components $0\leq \Pi_{1},\Pi_{2},\Pi_{3}\leq 1$ satisfy equality (21). These three probabilities are related to the superposition parameters as follows:

(26) $$\cos\xi =\frac{\Pi_{1}-1/2}{\sqrt{\Pi_{3}\left(1-\Pi_{3}\right)}}\;,\;\sin\xi =\frac{\Pi_{2}-1/2}{\sqrt{\Pi_{3}\left(1-\Pi_{3}\right)}}\;.$$

The three components of the vector ${\vec{P}}^{\left(3\right)}$ are functions of the three probability vectors ${\vec{P}}_{1}$, ${\vec{P}}_{2}$, and $\vec{\Pi }$; they read [23,24,48]

(27) $$\begin{array}{c} p_{3}^{\left(3\right)}=\Pi_{3}p_{3}^{\left(1\right)}+\left(1-\Pi_{3}\right)p_{3}^{\left(2\right)}+2\sqrt{p_{3}^{\left(2\right)}p_{3}^{\left(2\right)}}\left(\Pi_{1}-1/2\right),\\ p_{1}^{\left(3\right)}-1/2=\Pi_{3}\left(p_{1}^{\left(1\right)}-1/2\right)+\left(p_{1}^{\left(2\right)}-1/2\right)\left(1-\Pi_{3}\right)\\ +\left[\left(\Pi_{1}-1/2\right)\left(p_{1}^{\left(1\right)}-1/2\right)+\left(\Pi_{2}-1/2\right)\left(p_{2}^{\left(1\right)}-1/2\right)\right]\sqrt{p_{3}^{\left(2\right)}/p_{3}^{\left(1\right)}} \end{array}$$

(28) $$\begin{array}{c} +\left[\left(\Pi_{1}-1/2\right)\left(p_{1}^{\left(2\right)}-1/2\right)-\left(\Pi_{2}-1/2\right)\left(p_{2}^{\left(2\right)}-1/2\right)\right]\sqrt{p_{3}^{\left(1\right)}/p_{3}^{\left(2\right)}}, \end{array}$$

and the third one $p_{2}^{\left(3\right)}$ is determined in view of Equation (21).

As an example of the superposition of vectors $\frac{1}{\sqrt{2}}\left(\begin{array}{c} 1\\ 1 \end{array}\right)=|\psi_{1}\rangle$ and $\frac{1}{\sqrt{2}}\left(\begin{array}{c} 1\\ -1 \end{array}\right)=|\psi_{2}\rangle$ given by (24) and described by the probabilities (1,1/2,1/2) and (0,1/2,1/2), respectively, we obtain $p_{3}^{\left(3\right)}=\Pi_{1}$ and $p_{1}^{\left(3\right)}=\Pi_{3}$.

One can check that the obtained numbers $p_{1}^{\left(3\right)}$, $p_{2}^{\left(3\right)}$, and $p_{3}^{\left(3\right)}$ are nonnegative; they satisfy equality (21), which determines the probabilities $p_{2}^{\left(3\right)}$ and $\left(1-p_{2}^{\left(3\right)}\right)$. The unitary evolution of the probabilities $p_{1}$, $p_{2}$, and $p_{3}$ is described by the following transform of the matrix ρ (20)

(29) $$\rho \to u\rho u^{†},$$

where the unitary 2×2 matrix *u* is such that $u^{†}=u^{-1}$ and

(30) $$u\left(t\right)=e^{-iHt}=\left(\begin{array}{cc} u_{11}\left(t\right) & u_{12}\left(t\right)\\ u_{21}\left(t\right) & u_{22}\left(t\right) \end{array}\right),$$

with the Hamiltonian $H=\left(\begin{array}{cc} H_{11} & H_{12}\\ H_{21} & H_{22} \end{array}\right).$ From this evolution, which corresponds to the von Neumann equation

(31) $$\frac{\partial \rho }{\partial t}+i\left[H,\rho \right]=0,$$

the evolution formula for the probabilities follows; it reads

(32) $$\left(\begin{array}{c} p_{3}\left(t\right)\\ p_{1}\left(t\right)-\left(1/2\right)-i\left(p_{2}\left(t\right)-1/2\right)\\ p_{1}\left(t\right)-\left(1/2\right)+i\left(p_{2}\left(t\right)-1/2\right)\\ 1-p_{3}\left(t\right) \end{array}\right)=\left[u\left(t\right)⊗u^{*}\left(t\right)\right]\left(\begin{array}{c} p_{3}\\ p_{1}-\left(1/2\right)-i\left(p_{2}-1/2\right)\\ p_{1}-\left(1/2\right)+i\left(p_{2}-1/2\right)\\ 1-p_{3} \end{array}\right).$$

This result is the solution of Equation (31) written as the kinetic equation for probabilities $p_{1}\left(t\right)$, $p_{2}\left(t\right)$, and $p_{3}\left(t\right)$. Equation (32) describes the temporal evolution of the initial probabilities $p_{1}$, $p_{2}$, and $p_{3}$, which convert at time *t* to probabilities $p_{1}\left(t\right)$, $p_{2}\left(t\right)$, and $p_{3}\left(t\right)$ satisfying relation (23).

## 5. Quantum Observables and Classical-Coin Random Variables for Qubit Systems

The quantum observable for spin-1/2 system is described by the Hermitian matrices $A=\left(\begin{array}{cc} A_{11} & A_{12}\\ A_{21} & A_{22} \end{array}\right).$ It is possible [34] to consider quantum statistical properties of this observable in the probability representation associating the matrix elements $A_{jk}$ with classical-like random variables. Introducing the notation $A_{12}=x-iy$, $A_{11}=z_{1}$, and $A_{22}=z_{2}$, we can rewrite the mean value of the observable 〈A〉 in the form

(33) $$\langle A\rangle =Tr\;\rho A={\langle x\rangle }_{\mathrm{cl}}+{\langle y\rangle }_{\mathrm{cl}}+{\langle z\rangle }_{\mathrm{cl}},$$

where

(34) $${\langle x\rangle }_{\mathrm{cl}}=p_{1}x+\left(1-p_{1}\right)\left(-x\right),\;{\langle y\rangle }_{\mathrm{cl}}=p_{2}y+\left(1-p_{2}\right)\left(-y\right),\;{\langle z\rangle }_{\mathrm{cl}}=p_{3}z_{1}+\left(1-p_{3}\right)z_{2}.$$

This form shows that the mean values of the observable *A* calculated using the standard formalism of quantum mechanics provide the connection with classical-like means of three dichotomic random variables $x_{\mathrm{cl}}$, $y_{\mathrm{cl}}$, and $z_{\mathrm{cl}}$, employing the values (x,−x), (y,−y), and $\left(z_{1},z_{2}\right)$, respectively.

The probability distributions for these values are given as ${\vec{P}}_{1}=\left(p_{1},1-p_{1}\right)$, ${\vec{P}}_{2}=\left(p_{2},1-p_{2}\right)$, and ${\vec{P}}_{3}=\left(p_{3},1-p_{3}\right)$, which determine the density matrix ρ of the qubit state. The quantum observable in the quantum suprematism representation has a classical analog.

We point out that the highest moments of the observable *A* given as $\langle A^{n}\rangle =Tr\;\rho A^{n}$, n=2,3,… are also expressed in terms of classical random variables $x_{\mathrm{cl}}$, $y_{\mathrm{cl}}$, and $z_{\mathrm{cl}}$, and the expressions reflect quantum correlations of classical-like random observables due to the nonnegativity condition of the density matrix (23). One can also associate the matrix elements of arbitrary qudit observables with artificial-coin random variables.

## 6. Quantum Suprematism Representation

**Figure 1:**
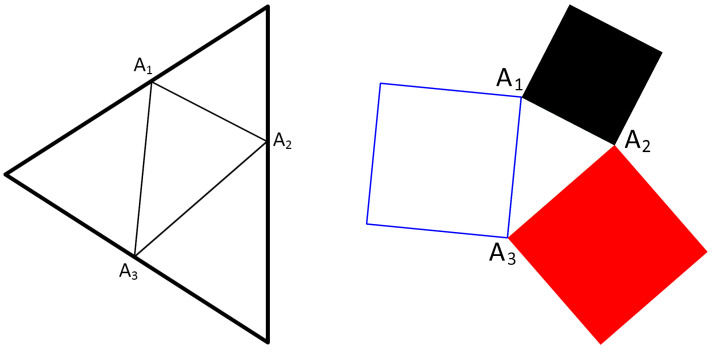
The equilateral triangle with a side length equal to $\sqrt{2}$. Each of three sides is simplex corresponding to the coin probabilities $p_{k}$ and $\left(1-p_{k}\right)$ satisfying the relation $p_{k}+\left(1-p_{k}\right)=1$; k=1,2,3. Here, the points on simplexes with probabilities $p_{1}$, $p_{2}$, and $p_{3}$ determine the triangle $A_{1}A_{2}A_{3}$ (**on the left**). The three squares (black, red, and white) called Triada of Malevich’s squares, which are constructed using the sides of the triangle $A_{1}A_{2}A_{3}$. The squares are in the one-to-one correspondence with the density matrix of qubit (spin-1/2) states (**on the right**).

The relations described in the previous section can be illustrated in the quantum suprematism picture [23,24,48], where the probabilities $p_{1}$, $p_{2}$, and $p_{3}$ determine the Triada of Malevich’s squares. We construct a triangle with vertices $A_{1}$, $A_{2}$, and $A_{3}$, which are located on three simplexes—sides of equilateral triangle with the side length equal to $\sqrt{2}$, and three squares (black, red, and white) determined by the sides of the triangle (Figure 1).

We call these squares the Triada of Malevich’s squares following [23,24,34,46,47]. The areas of the squares $S_{A_{1},A_{2}}$, $S_{A_{2},A_{3}}$, and $S_{A_{3},A_{1}}$ are

(35) $$\begin{array}{c} S_{A_{1},A_{2}}=2+2p_{2}^{2}-4p_{2}-2p_{3}+2p_{3}^{2}+2p_{2}p_{3},\\ S_{A_{2},A_{3}}=2+2p_{3}^{2}-4p_{3}-2p_{1}+2p_{1}^{2}+2p_{3}p_{1},\\ S_{A_{3},A_{1}}=2+2p_{1}^{2}-4p_{1}-2p_{2}+2p_{2}^{2}+2p_{1}p_{2}. \end{array}$$

The sum of the areas of Malevich’s squares, being the function of probabilities $p_{1}$, $p_{2}$, and $p_{3}$, reads

(36) $$S=2\left[3+2\left(p_{1}^{2}+p_{2}^{2}+p_{3}^{2}\right)-3\left(p_{1}+p_{2}+p_{3}\right)+p_{1}p_{2}+p_{2}p_{3}+p_{3}p_{1}\right].$$

The map of the Bloch sphere parameters $x_{k}$; k=1,2,3, onto the probabilities $p_{k}=\left(x_{k}+1\right)/2$ can be used to express the area *S* in terms of the parameters $x_{k}$.

For quantum states, the sum satisfies the inequality S≤3 [47]. For classical-coin states, the sum can take maximum value $S_{\mathrm{cl}}=6$. In view of this fact, the quantization condition (23) provides the possibility to clarify the difference of classical and quantum properties of the systems, which states are illustrated in the quantum suprematism representation by Triadas of Malevich’s squares.

The quantum states of spin-1/2 systems are described by spin tomogram $w(m|\vec{n})$ [16], and the spin tomogram determines the density matrix of the spin state [17]. The spin tomogram can be expressed by the probabilities $p_{1}$, $p_{2}$, and $p_{3}$; the tomogram is the probability to obtain the spin projection *m* onto the direction in space determined by the unit vector $\vec{n}=\left(\sin\theta \cos\varphi ,\sin\theta \sin\varphi ,\cos\theta \right)$.

For spin-1/2, the spin projection *m* takes two values ±1/2, and the tomogram (19) provides $w\left(m=+1/2|\vec{n}\right)=\vec{n}\left(\vec{p}-{\vec{p}}_{0}\right))+1/2$, where $\vec{p}=\left(\begin{array}{c} p_{1}\\ p_{2}\\ p_{3} \end{array}\right)$ and ${\vec{p}}_{0}=\left(\begin{array}{c} 1/2\\ 1/2\\ 1/2 \end{array}\right)$. The tomogram $w(m|\vec{n})$ can be interpreted as the conditional probability distribution and, in view of this fact, one can obtain a new entropic inequality associated with this distribution. For example, the relative Tsallis entropy for two distributions $w(m|{\vec{n}}_{1})$ and $w(m|{\vec{n}}_{2})$ satisfies the inequality, which yields the new condition for probabilities,

(37) $$\begin{array}{c} {\left(1-q\right)}^{-1}\left\{{\left[{\vec{n}}_{1}\left(\vec{p}-{\vec{p}}_{0}\right)+1/2\right]}^{q}{\left[{\vec{n}}_{2}\left(\vec{p}-{\vec{p}}_{0}\right)+1/2\right]}^{1-q}\right.\\ +\left.{\left[\left(1/2\right)-{\vec{n}}_{1}\left(\vec{p}-{\vec{p}}_{0}\right)\right]}^{q}{\left[\left(1/2\right)-{\vec{n}}_{2}\left(\vec{p}-{\vec{p}}_{0}\right)\right]}^{1-q}-1\right\}\geq 0. \end{array}$$

In the limit q→1, this inequality provides the nonnegativity condition for the von Neumann relative entropy

(38) $$\begin{array}{c} \left[{\vec{n}}_{1}\left(\vec{p}-{\vec{p}}_{0}\right)+1/2\right]\ln\left\{\left[{\vec{n}}_{1}\left(\vec{p}-{\vec{p}}_{0}\right)+1/2\right]{\left[{\vec{n}}_{2}\left(\vec{p}-{\vec{p}}_{0}\right)+1/2\right]}^{-1}\right\}\\ +\left[\left(1/2\right)-{\vec{n}}_{1}\left(\vec{p}-{\vec{p}}_{0}\right)\right]\ln\left\{\left[\left(1/2\right)-{\vec{n}}_{1}\left(\vec{p}-{\vec{p}}_{0}\right)\right]{\left[\left(1/2\right)-{\vec{n}}_{2}\left(\vec{p}-{\vec{p}}_{0}\right)\right]}^{-1}-1\right\}\geq 0. \end{array}$$

The obtained new entropic inequalities for probabilities $p_{1}$, $p_{2}$, and $p_{3}$ can be checked in the experiments with superconducting qubits, as well as the maximum value S=3 for the sum of areas of Malevich’s squares.

## 7. Qutrit States in the Probability Representation

One can extend the consideration of suprematism representation to the case of any qudits, for example, qutrit states with the density matrix $\rho =\left(\begin{array}{ccc} \rho_{11} & \rho_{12} & \rho_{13}\\ \rho_{21} & \rho_{22} & \rho_{23}\\ \rho_{31} & \rho_{32} & \rho_{33} \end{array}\right)$. Using the tool [23,24,47] of embedding this matrix into 4×4-matrix $R=\left(\begin{array}{cc} \rho & 0\\ 0 & 0 \end{array}\right)$, one can obtain three qubit-state density matrices applying the partial tracing procedure. These three-qubit density matrices are

(39) $$\rho \left(1\right)=\left(\begin{array}{cc} \rho_{11}+\rho_{22} & \rho_{13}\\ \rho_{31} & \rho_{33} \end{array}\right),\;\rho \left(2\right)=\left(\begin{array}{cc} \rho_{11}+\rho_{33} & \rho_{12}\\ \rho_{21} & \rho_{22} \end{array}\right),\;\rho \left(3\right)=\left(\begin{array}{cc} \rho_{22} & \rho_{23}\\ \rho_{32} & \rho_{11}+\rho_{33} \end{array}\right).$$

Since these matrices can be expressed in terms of probabilities

(40) $$\rho \left(k\right)=\left(\begin{array}{cc} p_{3}^{\left(k\right)} & p_{1}^{\left(k\right)}-\left(1/2\right)-i\left(p_{2}^{\left(k\right)}-1/2\right)\\ p_{1}^{\left(k\right)}-\left(1/2\right)+i\left(p_{2}^{\left(k\right)}-1/2\right) & 1-p_{3}^{\left(k\right)} \end{array}\right);\;k=1,2,3,$$

the qutrit density matrix elements can also be expressed in terms of these probabilities $p_{1,2,3}^{\left(k\right)}$. In fact, we arrive at

(41) $$\rho =\left(\begin{array}{ccc} p_{3}^{\left(1\right)}+p_{3}^{\left(2\right)}-1 & p_{1}^{\left(2\right)}-\left(1/2\right)-i\left(p_{2}^{\left(2\right)}-1/2\right) & p_{1}^{\left(1\right)}-\left(1/2\right)-i\left(p_{2}^{\left(1\right)}-1/2\right)\\ p_{1}^{\left(2\right)}-\left(1/2\right)+i\left(p_{2}^{\left(2\right)}-1/2\right) & 1-p_{3}^{\left(2\right)} & p_{1}^{\left(3\right)}-\left(1/2\right)-i\left(p_{2}^{\left(3\right)}-1/2\right)\\ p_{1}^{\left(1\right)}-\left(1/2\right)+i\left(p_{2}^{\left(1\right)}-1/2\right) & p_{1}^{\left(3\right)}-\left(1/2\right)+i\left(p_{2}^{\left(3\right)}-1/2\right) & 1-p_{3}^{\left(1\right)} \end{array}\right);$$

in other notation, Equation (41) is given in Appendix B. The probabilities $p_{1,2,3}^{\left(k\right)}$ satisfy inequality (23) for k=1,2,3 and also the nonnegativity condition for det ρ≥0. The expression of matrix elements of the qutrit density matrix in terms of the probability distributions follows a new entropic inequality for the matrix elements:

(42) $$\begin{array}{c} \frac{1}{1-q}\left\{{\left[\frac{1}{2}\left(\rho_{13}+\rho_{31}+1\right)\right]}^{q}{\left[\frac{1}{2}\left(\rho_{13}+\rho_{31}+1\right)\right]}^{1-q}\right.\\ +\left.{\left[\frac{1}{2}\left(-\rho_{13}-\rho_{31}+1\right)\right]}^{q}{\left[\frac{1}{2}\left(1-\rho_{23}-\rho_{32}\right)\right]}^{1-q}-1\right\}\geq 0. \end{array}$$

This new inequality for the qutrit state comes from applying the nonnegativity condition of the Tsallis entropy expressed in terms the qubit-state tomogram by Equations (37) and (38) to the qutrit-state density matrix.

Now, we derive the other inequality for the probabilities determining the qutrit state (41). First, we construct the qubit density matrices following [49]

(43) $$\begin{array}{c} \rho \left(2\right)=\left(\begin{array}{cc} p_{3}^{\left(2\right)} & p_{1}^{\left(2\right)}-\left(1/2\right)-i\left(p_{2}^{\left(2\right)}-1/2\right)\\ p_{1}^{\left(2\right)}-\left(1/2\right)+i\left(p_{2}^{\left(2\right)}-1/2\right) & 1-p_{3}^{\left(2\right)} \end{array}\right), \end{array}$$

(44) $$\begin{array}{c} \rho \left(2\right)=\left(\begin{array}{cc} p_{3}^{\left(1\right)} & p_{1}^{\left(1\right)}-\left(1/2\right)-i\left(p_{2}^{\left(1\right)}-1/2\right)\\ p_{1}^{\left(1\right)}-\left(1/2\right)+i\left(p_{2}^{\left(1\right)}-1/2\right) & 1-p_{3}^{\left(1\right)} \end{array}\right). \end{array}$$

The subadditivity condition provides the inequality for the probabilities $p_{1,2,3}^{\left(k\right)}$, k=1,2,3; it reads

(45) −Trρ(1)lnρ(1)−Trρ(2)lnρ(2)≥−Tr(ρlnρ),

where ρ, being given by (41), is determined by the probabilities.

In addition, the probabilities determining the qutrit states satisfy the Tsallis entropic inequality for the distance between the states

(46) $$\frac{1}{1-q}\left(Tr\;\rho {\left(1\right)}^{q}\rho {\left(2\right)}^{1-q}-1\right)\geq 0.$$

Inequalities (42), (45), and (46) are compatible with the nonnegativity condition of the qutrit density matrix.

Since the qubit density matrices (43) and (44) are obtained from the same qutrit density matrix, the distance between these qubit states characterizes the hidden correlations between the artificial qubits associated with the qutrit density matrix (41). The inequality can be checked in the experiments where the tomography of qutrit states is performed, e.g., in the experiments with superconducting circuits based on Josephson junction devices [50,51].

Since the qutrit states are described by the probabilities determining the states of three artificial qubits, the density matrix (39) can be mapped onto the set of three Triadas of Malevich’s squares.

## 8. Pure Qutrit States and Their Superposition in the Probability Representation

In [52,53], the superposition principle of quantum states was formulated as a nonlinear addition rule of the pure-state density operators. Namely, given two density operators ${\hat{\rho }}_{1}=|\psi_{1}\rangle \langle \psi_{1}|$ and ${\hat{\rho }}_{2}=|\psi_{2}\rangle \langle \psi_{2}|$ satisfying the conditions ${\hat{\rho }}_{1}^{2}={\hat{\rho }}_{1}$, ${\hat{\rho }}_{2}^{2}={\hat{\rho }}_{2}$, $Tr\;{\hat{\rho }}_{1}=Tr\;{\hat{\rho }}_{2}=1$, and ${\hat{\rho }}_{1}{\hat{\rho }}_{2}=0$. Then, for arbitrary real numbers $0\leq \lambda_{1},\lambda_{2}\leq 1$; $\lambda_{1}+\lambda_{2}=1$ and the density operator ${\hat{\rho }}_{0}=|\psi_{0}\rangle \langle \psi_{0}|$; ${\hat{\rho }}_{0}^{2}={\hat{\rho }}_{0}$, the state with the density operator ${\hat{\rho }}_{\psi }$ of the form

(47) $${\hat{\rho }}_{\psi }=\lambda_{1}{\hat{\rho }}_{1}+\lambda_{2}{\hat{\rho }}_{2}+\sqrt{\lambda_{1}\lambda_{2}}\;\;\frac{{\hat{\rho }}_{1}{\hat{\rho }}_{0}{\hat{\rho }}_{2}+{\hat{\rho }}_{2}{\hat{\rho }}_{0}{\hat{\rho }}_{1}}{\sqrt{Tr\left({\hat{\rho }}_{1}{\hat{\rho }}_{0}{\hat{\rho }}_{2}{\hat{\rho }}_{0}\right)}}$$

satisfies the conditions ${\hat{\rho }}_{\psi }^{†}={\hat{\rho }}_{\psi }$, ${\hat{\rho }}_{\psi }^{2}={\hat{\rho }}_{\psi }$, and $Tr\;{\hat{\rho }}_{\psi }=1$.

The nonlinear addition rule (47) corresponds to the interference formula of two orthogonal pure states $|\psi_{1}\rangle$ and $|\psi_{2}\rangle$ of the form $|\psi \rangle =\sqrt{\lambda_{1}}|\psi_{1}\rangle +e^{i\varphi }\sqrt{\lambda_{2}}|\psi_{2}\rangle$, where the phase φ is coded by an artificial density operator ${\hat{\rho }}_{0}$.

One can use generic Equation (47) to formulate the superposition rule of qudit states expressed in terms of the probabilities; we obtain such a formula for two qutrit states. For this, we introduce three probability vectors, i.e., three probability distributions $\vec{\Pi_{1}}=\left(\Pi_{1},1-\Pi_{1}\right)$, $\vec{\Pi_{2}}=\left(\Pi_{2},1-\Pi_{2}\right)$, and $\vec{\Pi_{3}}=\left(\Pi_{3},1-\Pi_{3}\right)$, where $0\leq \Pi_{1},\Pi_{2},\Pi_{3}\leq 1$, and the phase φ is defined by the relations (26),

(48) $$\cos\varphi =\frac{\Pi_{1}-1/2}{\sqrt{\Pi_{3}\left(1-\Pi_{3}\right)}}\;,\;\sin\varphi =\frac{\Pi_{2}-1/2}{\sqrt{\Pi_{3}\left(1-\Pi_{3}\right)}}\;.$$

This means that we arrive at the condition ${\left(\Pi_{1}-1/2\right)}^{2}+{\left(\Pi_{2}-1/2\right)}^{2}+{\left(\Pi_{3}-1/2\right)}^{2}=1/4$ equivalent to the condition (21). To obtain the superposition rule for qutrit states in the probability representation, we employ the expression of density matrix (41), introduce three 8-vectors ${\vec{P}}_{1}$, ${\vec{P}}_{2}$, and ${\vec{P}}_{0}$ of the form (see Appendix B):

(49) $$\begin{array}{c} {\vec{P}}_{1}=\left(p_{1}^{\left(I\right)},p_{2}^{\left(I\right)},p_{3}^{\left(I\right)},p_{4}^{\left(I\right)},p_{5}^{\left(I\right)},p_{6}^{\left(I\right)},p_{7}^{\left(I\right)},p_{8}^{\left(I\right)}\right),\\ {\vec{P}}_{2}=\left(p_{1}^{\left(\mathrm{II}\right)},p_{2}^{\left(\mathrm{II}\right)},p_{3}^{\left(\mathrm{II}\right)},p_{4}^{\left(\mathrm{II}\right)},p_{5}^{\left(\mathrm{II}\right)},p_{6}^{\left(\mathrm{II}\right)},p_{7}^{\left(\mathrm{II}\right)},p_{8}^{\left(\mathrm{II}\right)}\right),\\ {\vec{P}}_{0}=\left(p_{1}^{\left(\mathrm{III}\right)},p_{2}^{\left(\mathrm{III}\right)},p_{3}^{\left(\mathrm{III}\right)},p_{4}^{\left(\mathrm{III}\right)},p_{5}^{\left(\mathrm{III}\right)},p_{6}^{\left(\mathrm{III}\right)},p_{7}^{\left(\mathrm{III}\right)},p_{8}^{\left(\mathrm{III}\right)}\right), \end{array}$$

and identify three qutrit states ${\hat{\rho }}_{1}$, ${\hat{\rho }}_{2}$, and ${\hat{\rho }}_{3}$ with these vectors ${\vec{P}}_{1}$, ${\vec{P}}_{2}$, and ${\vec{P}}_{0}$, since the density matrices are determined by their components. Formula (47) provides the dependence of the probability vector ${\vec{P}}_{\psi }$, which determines the state ${\hat{\rho }}_{\psi }$, on the probabilities ${\vec{P}}_{1}$, ${\vec{P}}_{2}$, ${\vec{P}}_{0}$, and ${\vec{\Pi }}_{1,2,3}$. The density matrix (41) of the qutrit pure state satisfies the condition $\rho^{2}=\rho$, which provides the formula for the probabilities following from the equality of the matrix elements $\sum_{k}\rho_{jk}\rho_{km}=\rho_{jm}$ written in terms of probabilities.

Thus, we formulated the result for qutrits. We rewrite Equation (47) expressing the matrices ${\hat{\rho }}_{\psi }$, ${\hat{\rho }}_{1}$, ${\hat{\rho }}_{2}$, and ${\hat{\rho }}_{0}$ in terms of probabilities. The equality of matrix elements provides the expression of the probability vectors ${\vec{P}}_{\psi }$ as functions of the probability vectors ${\vec{P}}_{1}$, ${\vec{P}}_{2}$, ${\vec{P}}_{0}$, and $\Pi_{1,2,3}$. The approach can be extended to other qudit states.

## 9. Probability Representation of the Density *N*×*N*-Matrix of the Qudit State

We generalize Equation (41) and write the matrix element $\rho_{jk}$ for an arbitrary qudit state (*N*-level atom state) in the form [48]:

(50) $$\begin{array}{c} \rho_{jk}=p_{1}^{\left(jk\right)}-\left(1/2\right)-i\left(p_{2}^{\left(jk\right)}-1/2\right),\;k>j,\\ \rho_{jj}=1-p_{3}^{\left(jj\right)},\;j\geq 2,\\ \rho_{22}=1-\sum_{j=2}^{N}\rho_{jj}, \end{array}$$

where $p_{1}^{\left(jk\right)}$ and $p_{2}^{\left(jk\right)}$ are the probabilities for artificial qubits (spin-1/2 states) to have spin projections m=+1/2 onto the *x* and *y* axes, respectively. The diagonal matrix elements $\rho_{jj}$ depend on probabilities $p_{3}^{\left(jj\right)}$, which are probabilities of artificial spin projections m=+1/2 on the *z*-axis. Thus, all the matrix elements of the qudit density matrix are expressed in terms of probabilities for $\left(N^{2}-1\right)$ classical coins to have positions “UP” or “DOWN.” The new inequality (42) is also valid for the qudit density matrix (50) as well as the entropic inequalities for the matrix elements where 1→j and 3→k.

The superposition principle of pure states given by relation (47) for the density matrices provides the connection of the probabilities determining two orthogonal pure states ${\hat{\rho }}_{1}$ and ${\hat{\rho }}_{2}$ and the density matrix ${\hat{\rho }}_{0}$ with the probabilities determining the pure superposition state ${\hat{\rho }}_{\psi }$. In spite of the fact that the formulas are cumbersome, their existence demonstrates that such quantum phenomenon as interference of quantum states for arbitrary *N*-level atoms can be formulated as nonlinear superposition of classical probabilities (cf. [30,31,32]). Thus, for any qudit state, one has relationships connecting the probability distributions determining the qudit states in view of generic formula (47).

## 10. Conclusions

To conclude, we point out the main results of our study.

We introduced the notion of hidden correlations for systems without subsystems, using the explicitly written functions (5)–(7) and (9)–(12) providing the invertible map of the integers in both classical and quantum domains. This approach provides the possibility to write Bayes’ formula and introduce the conditional probability distribution for given probability distribution of one random variable (22). Using the probability description of qubit states, we presented the solution of the von Neumann equation for the two-level atom as the transform of probabilities determining the state density matrix (35).

We obtained new inequality for the qubit-state probabilities, which can be checked experimentally as the nonnegativity condition of classical relative Tsallis entropy—Equation (38). The properties of generalized entropies discussed and employed in [21,22] can be also studied in view of the probability distributions determining the matrix elements of the density matrix. We will do this in future publications.

The tomographic reconstruction of the density matrix, e.g., in the experiments with superconductive circuits [50,51], provides the possibility to find the probabilities that should satisfy the inequalities.

In view of a generic form of the superposition principle formulated in terms of density matrices (47), we presented the general approach to get the addition rule for classical probabilities determining the qutrit pure state. In this sense, we considered two related problems. One problem is to reformulate the standard description of quantum mechanics by means of wave functions and density matrices, using the Hilbert space formalism, in terms of classical probabilities; this can be done within the framework of quantum tomography [15,16,17,29]. The inverse approach is to consider classical probabilities associated with quantumlike objects in Hilbert spaces [30,31,32]. In this paper, we concentrate on the presentation of the first problem and show that the quantum formalism of Hilbert spaces can be mapped bijectively onto the classical-like formalism of the probability theory and geometry of simplexes. New results obtained in this approach are the nonlinear addition rules of probabilities, giving the probabilities. Such rules correspond either to Born rules of quantum mechanics or the superposition principle of pure states of qudits.

In addition, we obtained an explicit form of the unitary evolution for probabilities determining the probabilities in terms of their transform by means of unitary matrices for two-level atoms.

To illustrate the map of Bloch sphere parametrization of qubit states onto the probability representation of the states, where the probabilities satisfy the quantum constraint inequalities, we employed the geometric representation of the probabilities, in view of the suprematism picture of Triadas of Malevich’ squares. The approach developed and its properties in the case of generic qudit states will be elaborated in the future publication.

In addition, we point out that the tomographic-probability approach was applied in signal analysis [54,55] due to the descriptions of the signals by an analog of the Wigner function proposed by Ville [56]. The probability properties considered above can be used in the signal theory as well.

## Appendix A

We consider a simple example of N=4 and $n_{1}=n_{2}=2$. We have the distribution P(j,k) of four numbers 1≥P(1,1),P(1,2),P(2,1),P(2,2)≥0. In view of Equations (2) and (3), we have the conditional probabilities:

$$\begin{array}{c} P\left(1|1\right)=\frac{P(1,1)}{P(1,1)+P(2,1)}\;,\;P\left(2|1\right)=\frac{P(2,1)}{P(1,1)+P(2,1)}\;,\\ P\left(1|2\right)=\frac{P(1,2)}{P(1,2)+P(2,2)},\;P\left(2|2\right)=\frac{P(2,2)}{P(1,2)+P(2,2)}\;. \end{array}$$

Numbers P(1∣1), P(2∣1), P(1∣2), and P(2∣2) are nonnegative and normalized, $\sum_{j=1}^{2}P\left(j|k\right)=1$.

Now, we consider the example of the same four nonnegative numbers, which we denote as P(n), with n=1,2,3,4, and define the numbers as P(1)=P(1,1), P(2)=P(2,1), P(3)=P(1,2), and P(4)=P(2,2). The set of numbers P(n) can be interpreted as the probability distribution of one random variable *n*. Since Bayes’ formula is determined through only the numbers P(j,k), we can apply it using the invertible map of integers 1↔1,1, 2↔2,1, 3↔1,2, 4↔2,2 and introducing two “marginal probability distributions” $P_{1}\left(1\right)=P\left(1,1\right)+P\left(1,2\right)=P\left(1\right)+P\left(3\right)$, $\;P_{1}\left(2\right)=P\left(2,1\right)+P\left(2,2\right)=P\left(2\right)+P\left(4\right)$ and $\;P_{2}\left(1\right)=P\left(1,1\right)+P\left(2,1\right)=P\left(1\right)+P\left(2\right)$, $P_{2}\left(2\right)=P\left(1,2\right)+P\left(2,2\right)=P\left(3\right)+P\left(4\right)$. After this, we can introduce the “conditional probability distributions” in view of the definition given by the same relations (3) and (4) but rewritten in terms of numbers P(n), with n=1,2,3,4. We define the conditional probabilities

$$\begin{array}{c} P\left(1|1\right)=\frac{P(1)}{P(1)+P(3)}\;,\;P\left(2|1\right)=\frac{P(2)}{P(1)+P(3)}\;,\\ P\left(1|2\right)=\frac{P(3)}{P(2)+P(4)}\;,\;P\left(2|2\right)=\frac{P(4)}{P(2)+P(4)}\;. \end{array}$$

## Appendix B

To express the qutrit density matrix ρ in the probability representation, we employ the 8-vector $\vec{P}=\left(p_{1},p_{2},p_{3},p_{4},p_{5},px_{6},p_{7},p_{8}\right)$ and obtain

(A1) $$\rho =\left(\begin{array}{ccc} p_{3}+p_{6}-1 & p_{4}-\left(1/2\right)-i\left(p_{5}-1/2\right) & p_{1}-\left(1/2\right)-i\left(p_{2}-1/2\right)\\ p_{4}-\left(1/2\right)+i(p_{5}-\left(1/2\right) & 1-p_{6} & p_{7}-\left(1/2\right)-i(p_{8}-\left(1/2\right)\\ p_{1}-\left(1/2\right)+i\left(p_{2}-1/2\right) & p_{7}-\left(1/2\right)+i\left(p_{8}-1/2\right) & 1-p_{3} \end{array}\right).$$

We have the probability $p_{9}=1-p_{6}$, and nine probability distributions, corresponding to classical-coin positions, read ${\vec{p}}_{j}=\left(p_{j},1-p_{j}\right)$; j=1,2,…,8 and ${\vec{p}}_{9}=\left(p_{9},1-p_{9}\right)=\left(1-p_{6},p_{6}\right)$. Thus, the density matrix of qutrit state formally corresponds to the probability distributions describing positions "UP" and "DOWN" of nine classical coins. In our notation, the ninth coin probability distribution is completely correlated with the sixth coin position, i.e., it is determined by the probability distribution of the sixth coin. The other correlations are described by nonnegativity conditions for the density-matrix eigenvalues. The density matrices $\rho_{1}$, $\rho_{2}$, and $\rho_{0}$ have the form (A1) with the replacement $p_{j}\to p_{j}^{\left(I\right)},p_{j}^{\left(\mathrm{II}\right)},p_{j}^{\left(\mathrm{III}\right)}$; j=1,2,…,8.

The matrix $\rho_{0}$ is determined by the probability vector ${\vec{P}}_{0}=\left\{p_{j}^{\left(0\right)}\right\}$; j=1,2,…,8. All of the 8-vectors ${\vec{P}}_{0}$, ${\vec{P}}_{1}$, and ${\vec{P}}_{2}$ satisfy the condition that the corresponding density matrices satisfy the equality $Tr\;{\hat{\rho }}_{1}^{2}=Tr\;{\hat{\rho }}_{2}^{2}=Tr\;{\hat{\rho }}_{0}^{2}=1$ and the property $Tr\;{\hat{\rho }}_{\psi }^{2}=1$ follows relation (47).
